# Novel Scalable and Simplified System to Generate Microglia-Containing Cerebral Organoids From Human Induced Pluripotent Stem Cells

**DOI:** 10.3389/fncel.2021.682272

**Published:** 2021-07-05

**Authors:** Brittany Bodnar, Yongang Zhang, Jinbiao Liu, Yuan Lin, Peng Wang, Zhengyu Wei, Sami Saribas, Yuanjun Zhu, Fang Li, Xu Wang, Wenli Yang, Qingsheng Li, Wen-Zhe Ho, Wenhui Hu

**Affiliations:** ^1^Department of Pathology and Laboratory Medicine, Temple University Lewis Katz School of Medicine, Philadelphia, PA, United States; ^2^Center for Metabolic Disease Research, Temple University Lewis Katz School of Medicine, Philadelphia, PA, United States; ^3^Center for Stem Cell Research and Application, Institute of Blood Transfusion, Chinese Academy of Medical Sciences and Peking Union Medical College (CAMS and PUMC), Chengdu, China; ^4^Institute for Regenerative Medicine and Department of Medicine, University of Pennsylvania, Philadelphia, PA, United States; ^5^Nebraska Center for Virology, School of Biological Sciences, University of Nebraska-Lincoln, Lincoln, NE, United States

**Keywords:** cerebral organoids, microglia-containing cerebral organoids, induced pluripotent stem cells, neural induction, human brain development

## Abstract

Human cerebral organoid (CO) is a three-dimensional (3D) cell culture system that recapitulates the developing human brain. While CO has proved an invaluable tool for studying neurological disorders in a more clinically relevant matter, there have still been several shortcomings including CO variability and reproducibility as well as lack of or underrepresentation of certain cell types typically found in the brain. As the technology to generate COs has continued to improve, more efficient and streamlined protocols have addressed some of these issues. Here we present a novel scalable and simplified system to generate microglia-containing CO (MCO). We characterize the cell types and dynamic development of MCOs and validate that these MCOs harbor microglia, astrocytes, neurons, and neural stem/progenitor cells, maturing in a manner that reflects human brain development. We introduce a novel technique for the generation of embryoid bodies (EBs) directly from induced pluripotent stem cells (iPSCs) that involves simplified steps of transitioning directly from 3D cultures as well as orbital shaking culture in a standard 6-well culture plate. This allows for the generation of MCOs with an easy-to-use system that is affordable and accessible by any general lab.

## Introduction

The brain is a complex organ consisting of intricate neural networks with extensive functions. Understanding the cellular and molecular mechanisms underlying neurodevelopmental disorders and neurological/neuropsychiatric diseases has been complicated by the limited availability of clinical tissues and animal models to fully reflect the human brain. In the last decade, advances in stem cell technology have led to the use of human-induced pluripotent stem cells (iPSCs) to generate three-dimensional (3D) models termed cerebral organoids (COs; Lancaster et al., 2013). Within COs, cells self-organize into 3D tissues and differentiate into multiple cell types, recapitulating many features of human neurodevelopment (Amiri et al., 2018; Velasco et al., 2019). Because human iPSCs can be readily generated from any cell type using advanced reprogramming techniques, COs have been extensively utilized to help uncover pathogenic mechanisms and guide drug discovery in a number of neurological diseases and disorders. These applications include neurodevelopmental disorders such as autism spectrum disorders and related disorders (Mariani et al., 2015; Chailangkarn et al., 2016; Birey et al., 2017; Wang et al., 2017; Mellios et al., 2018; Gouder et al., 2019; Sun et al., 2019), neuropsychiatric disorders such as schizophrenia (Srikanth et al., 2015; Stachowiak et al., 2017; Ye et al., 2017; De Vrij et al., 2019; Kathuria et al., 2020), structural and migration disorders such as microencephaly and lissencephaly (Lancaster et al., 2013; Bershteyn et al., 2017; Iefremova et al., 2017; Li et al., 2017a,b; Fiddes et al., 2018; Karzbrun et al., 2018; Zhang et al., 2019), and even neurotropic viral infections such as Zika Virus (Cugola et al., 2016; Dang et al., 2016; Qian et al., 2016; Gabriel et al., 2017; Li et al., 2017b; Watanabe et al., 2017) and SARS-CoV-2 (Jacob et al., 2020; Pellegrini et al., 2020a; Ramani et al., 2020; Yang et al., 2020; Zhang et al., 2020).

Since the inception of COs, there have been rapid and significant advancements in organoid culture technology, increasing their accessibility and reproducibility. Original protocols for COs require the use of expensive and bulky equipment and a large volume of reagents, as spinning culture is necessary for enhanced nutrient availability (Lancaster et al., 2013; Lancaster and Knoblich, 2014); newer protocols have utilized innovative methods such as 3D-printed spinning bioreactors (Qian et al., 2016, 2018), allowing for increased efficiency and larger-scale production of COs. Many studies have incorporated the directed differentiation to generate various brain region-specific COs, including the midbrain (Jo et al., 2016; Qian et al., 2016, 2018; Monzel et al., 2017; Jacob et al., 2020), hippocampus (Sakaguchi et al., 2015; Jacob et al., 2020), choroid plexus (Sakaguchi et al., 2015; Jacob et al., 2020; Pellegrini et al., 2020a,b), thalamus (Xiang et al., 2019, 2020), ganglionic eminence (Bagley et al., 2017; Birey et al., 2017; Watanabe et al., 2017; Xiang et al., 2017, 2018), hypothalamus/pituitary (Suga et al., 2011; Ogawa et al., 2018; Qian et al., 2018; Kasai et al., 2020; Matsumoto et al., 2020), and cerebellum (Muguruma et al., 2015; Holmes and Heine, 2017; Watson et al., 2018). Further innovations include the fusion of region-specific COs to generate “brain assembloids,” which are particularly useful for studying interneuron migration and neuronal projections between organoid structures (Bagley et al., 2017; Birey et al., 2017; Xiang et al., 2017, 2018, 2019; Sloan et al., 2018; Wörsdörfer et al., 2019).

In order to most accurately reflect neurodevelopment, it is important that all cell types within the developing human brain are proportionally represented as closely as possible. However, one of the current limitations for the original CO protocols is not all these cell types are readily generated, particularly for cell types of non-ectodermal origin. Since microglia derives from mesoderm while neural stem cells (NSCs)/neural progenitor cells (NPCs) derive from neuroepiderm, early studies had to culture iPSC-derived microglia and NSCs/NPCs separately first and then co-culture them (Schwartz et al., 2015; Muffat et al., 2016, 2018; Abud et al., 2017; Douvaras et al., 2017; Haenseler et al., 2017; Garcia-Reitboeck et al., 2018; Song et al., 2019; Wörsdörfer et al., 2019). Most recent studies documented that non-inhibition of bone morphogenetic protein signaling during CO culture allowed the generation of mesoderm-derived progenitor cells within COs (Quadrato et al., 2017; Ormel et al., 2018). Of interest, one study modified neural induction condition by reducing heparin concentration, rendering the innate generation of mesodermal cells, which readily differentiated into microglial cells within the CO, named MCO (Ormel et al., 2018).

Following this approach for inclusion of innate microglia that plays an essential role in brain development, neural innate immunity, neuroinflammation, and neurotropic viral infection, we developed a novel scalable and simplified protocol for the generation of MCO from human iPSCs. Most CO protocols require complicated systems involving manual selection of iPSC colonies and growing in aggregates in droplet suspension (Amiri et al., 2018; Ormel et al., 2018; Velasco et al., 2019). Our protocol utilizes a novel method for direct transition from iPSC aggregate 3D culture to embryoid bodies (EBs), which has not been previously reported. Furthermore, our system uses six-well plates on an orbital shaker for constant shaking culture, which are easily accessible materials for most labs. This also significantly reduces the amount of culture media needed for COs, further reducing cost. Here we validated the MCO model with an easy-to-use scalable protocol.

## Materials and Methods

### Generation and Expansion of Human Induced Pluripotent Stem Cells

Human primary fibroblasts from apparently healthy individuals were purchased from the Coriell Institute for Medical Research [GM00942 and GM00969]. These fibroblasts were used to generate human-induced pluripotent stem cells (hiPSCs) at the University of Pennsylvania’s iPSC Core using CytoTune 1.0 Sendai Virus reprogramming factors (Thermofisher Scientific) according to the manufacturer’s directions. After iPSC lines were established, they were transferred to and maintained in feeder-free conditions using mTeSR-Plus serum-free media (Stem Cell Technologies) on 6-well plates coated with 1:100 Matrigel hESC-qualified matrix (Corning), according to the manufacturer protocols. Cells were passaged when 70–90% confluent (around 3–5 days) using ReLeSR enzyme-free passaging reagent (Stem Cell Technologies). Cells were checked for karyotypic abnormalities every five passages using the iPSC Genetic Analysis Kit (Stem Cell Technologies), following manufacturer protocols. All iPSCs used for organoid generation were between passages 12 and 25. All cells and organoids were maintained in an incubator at 37°C and 5% CO_2_.

### Suspension 3D Culture of iPSCs

To generate 3D spheres, iPSCs were dissociated as clusters from Matrigel-coated plates using ReLeSR (Stem Cell Technologies) and resuspended in mTeSR-3D seed medium (Stem Cell Technologies) containing 10 μM Y-27632 ROCK inhibitor (Stem Cell Technologies). The dissociated iPSC clusters were then seeded on low-attachment 6-well plates and maintained on an orbital shaker (Benchmark Scientific, BT4001), shaking constantly at 65–70 rpm. The iPSCs aggregated to form 3D spheres in suspension culture and mTeSR 3D feed medium was added every day.

### Generation of Embryoid Bodies Directly From 3D Culture *via* Neural Induction for Microglia-Containing Cerebral Organoids (MCOs)

When 3D spheres were around 300–400 μm in diameter (around 4–5 days), they were collected in a 15-ml conical tube. The old medium was aspirated off and replaced with EB medium (Lancaster and Knoblich, 2014; Bagley et al., 2017), which consists of DMEM/F-12 (Corning), 20% KnockOut Serum Replacement (KOSR; Gibco), 3% hESC-quality FBS (Gibco), 1 mM GlutaMAX (Gibco), 0.1 mM minimal essential medium non-essential amino acids (MEM-NEAA; Gibco), 1% penicillin/streptomycin (Corning), and 0.1 mM 2-mercaptoethanol (Pierce). The iPSC 3D spheres in EB medium were cultured in low-attachment 6-well plates, at a density of 50–100 spheres per well to generate EB (considered day 0 of organoid culture) directly from 3D spheres. After 2 days (day 2), $\frac{3}{4}$ of the EB medium was refreshed. After 4 days of EB culture (Day 4), $\frac{3}{4}$ of the medium was changed every other day using neural induction medium (NIM; Ormel et al., 2018), which consists of DMEM/F-12, 1% N2 supplement (Gibco), 1 mM GlutaMAX, 0.1 mM MEM-NEAA, 1% penicillin/streptomycin, and 0.1 μg/ml heparin (Stem Cell Technologies), 10- to 100-fold lower than original CO culture protocols (Lancaster and Knoblich, 2014; Krefft et al., 2018; Yakoub and Sadek, 2018; Zhang et al., 2019), which is the critical step for the induction of innate microglia generation (Ormel et al., 2018).

### Matrigel Embedding and Organoid Maturation

On day 12, organoids were embedded in growth-factor reduced Matrigel (Corning), as described previously with modification (Qian et al., 2018). Briefly, organoids were collected to 15 ml conical tube and resuspended in 1 ml of cerebral organoid differentiation medium (CODM; Lancaster and Knoblich, 2014), which consists of a 1:1 ratio of DMEM/F-12 and Neurobasal medium (Gibco), 1% penicillin/streptomycin, 1% B27 supplement without vitamin A (Gibco), 0.5% N2 supplement, 2.5 μg/ml insulin, 0.05 mM MEM-NEAA, and 0.05 mM 2-mercaptoethanol. Using cut pipette tips, organoids in microcentrifuge tubes (~20–30 per tube) were resuspended with Matrigel (2:3) and spread onto low-attachment 6-well plates. Once solidified at 37°C for 30 min, 2 ml CODM was added to each well, and plates were kept in the incubator in non-spinning culture. On day 16, organoids were mechanically dissociated from the Matrigel and resuspended in CODM with vitamin A (CODMA), which is prepared the same way as CODM, but uses 1% B27 supplement with vitamin A (Gibco). Organoids were maintained in spinning culture, and media was changed every 3–4 days until experimental endpoints.

### Multilabeled Fluorescent Immunocytochemistry and Confocal Image Analysis

Organoids were fixed overnight using 4% paraformaldehyde (PFA). After fixation, organoids were incubated in 30% sucrose then embedded and frozen in optimal cutting tissue (OCT) medium. Frozen tissues were sectioned using cryostat at 10 μm thickness. Sections were permeabilized with 0.5% TritonX-100/1× phosphate-buffered saline (PBS) for 30 min, blocked with 10% donkey serum for 1 h, and incubated with primary antibodies (Table 1) in 0.1% TritonX-100/1× PBS overnight at 4°C. The next day, slides were washed with 1× PBS and incubated with the corresponding Alexa Fluor AffiniPure secondary antibodies (Jackson Immuno Research Labs; donkey anti-goat, anti-rabbit, anti-mouse, or anti-chicken IgG (H + L) 488, 594, or 680) at a 1:400 dilution for 1 h at room temperature, using Hoechst 33258 (1:5,000) as a nuclear counterstain. Slides were then coverslipped using Fluoroshield histology mounting medium (Sigma-Aldrich). Fluorescent confocal images were acquired and analyzed using the Leica SP8 confocal system.

**Table 1:** Primary antibodies used for immunohistochemistry.

Primary antibody	Species and clonality	Manufacturer	Dilution
Iba1	Rabbit polyclonal	Proteintech, cat. no. 10904–1-AP	1:200
Doublecortin	Goat polyclonal	Santa cruz biotechnology, cat. no. sc-8066	1:500
Tuj1	Chicken polyclonal	Aves, cat. no. TUJ	1:2,000
Nestin	Chicken polyclonal	Aves, cat. no. NES	1:200
GFAP	Chicken polyclonal	Aves, cat. no. GFAP	1:500
TMEM119 (extracellular)	Mouse monoclonal	BioLegend, cat. no. 853302	1:200
SOX2	Goat polyclonal	Santa cruz biotechnology, cat. no. sc-17320	1:200
mGluR5	Chicken polyclonal	Aves, cat. no. ER5	1:200
NeuN	Mouse monoclonal	Millipore-sigma, cat. no. MAB377	1:500
Ki67	Rabbit monoclonal	Abcam, cat. no. ab16667	1:400
PAX6	Mouse monoclonal	DSHB, RRID AB_528427	1:200
GAD67	Chicken polyclonal	Aves, cat. no. GAD	1:1,000
PSD95	Rabbit polyclonal	Proteintech, cat. no. 20665–1-AP	1:400
MAP2	Chicken polyclonal	Aves, cat. no. MAP	1:1,000
Synaptophysin	Mouse monoclonal	Dako, cat. no. M0776	1:1,000

*Note: The PAX6 mouse monoclonal antibody was deposited to the Developmental Studies Hybridoma Bank (DSHB) by Kawakami*.

### Flow Cytometry

Each MCO was dissociated to a single-cell suspension using Accutase (Corning), passed through a 70 μm nylon cell strainer (Corning) to remove large clumps, and washed with 1× PBS. Cells were centrifuged (500× *g* for 5 min) and resuspended in 500 μl cell staining buffer (BioLegend cat. no. 420201). Cells were incubated with the primary antibodies anti-CD11b-FITC (Biolegend cat. no. 301329) and anti-P2RY12-PE (Biolegend cat. no. 392103) in the dark for 30 min at room temperature. FITC mouse IgG1-κ (BD Biosciences cat. no. 551954) and PE mouse IgG2a-κ (BD Biosciences cat. no. 555574) were used as isotype controls. Following staining, cells were washed with cell staining buffer, centrifuged, and fixed with 4% paraformaldehyde in PBS. Analysis was performed using Cytek Aurora Flow cytometer.

### Statistical Analysis

Statistical analysis was performed using Prism GraphPad 9.1. Significance at *P* < 0.05 was determined between two groups of different time or markers using a two-tailed student’s *t*-test.

## Results

### Characterization of iPSCs Derived From Human Fibroblasts by Footprint-Free Sendai Virus Technology

**Figure 1:**
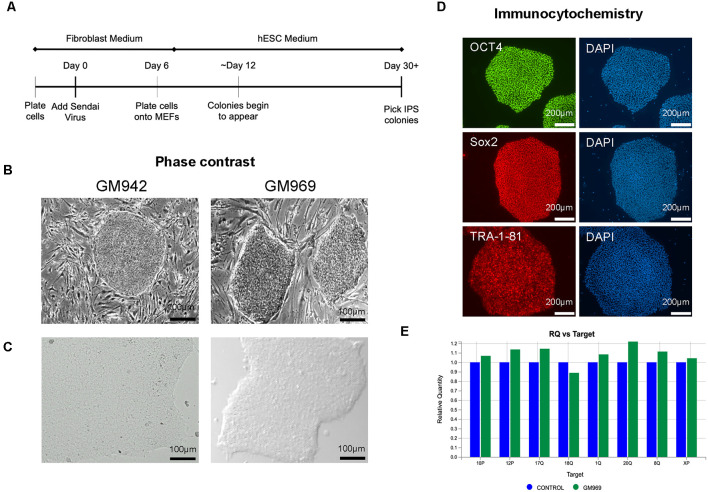
Generation and characterization of human-induced pluripotent stem cells (iPSCs). **(A)** Timeline of Sendai Virus reprogramming from human fibroblasts. **(B,C)** Phase-contrast imaging of iPSC colonies in feeder-dependent culture **(B)** and feeder-free culture **(C)**. **(D)** Confirmation of iPSC identity using pluripotency markers, OCT4, SOX2, and TRA-1–81, using DAPI to stain nuclei. **(E)** Karyotypic analysis of iPSCs confirming no genomic abnormalities.

To generate iPSCs, human fibroblasts from apparently healthy individuals were sent to the University of Pennsylvania iPSC Core for reprogramming using Sendai virus technology (Figure 1A) in MEF-feeder culture (Figure 1B). We then transitioned the iPSCs to feeder-free conditions by passaging 3–5 times using mTeSR-Plus medium and maintaining it as a monolayer culture (Figure 1C). The iPSC identity was confirmed using immunocytochemical staining for pluripotency markers, including OCT4, SOX2, and TRA-1–81 (Figure 1D). The iPSC lines were regularly screened for karyotypic abnormalities (Figure 1E) as quality control and maintained in feeder-free conditions for iPSC line expansion.

### Efficient Generation of MCOs Using Simple 3D Transition Protocol

**Figure 2:**
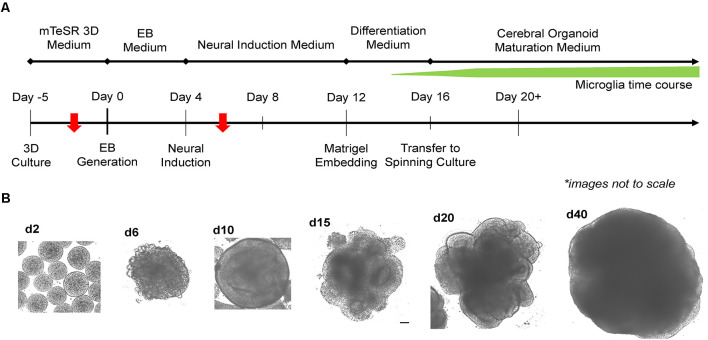
Experimental timeline for generation of microglia-containing cerebral organoids (MCOs) and representative images for each stage of MCO formation and maturation. **(A)** Schematic diagram for MCO generation from iPSC 3D culture. The red arrows indicate two key steps (EB initiation and innate microglia induction). **(B)** Representative phase-contrast images of MCOs at various stages of growth and maturation.

Two critical steps were combined to generate MCO. For EB formation, we expanded 2D iPSCs into 3D spheres in suspension culture using mTeSR-3D medium in a 6-well plate under 65–70 rpm in an orbital shaker (Lancaster and Knoblich, 2014; Qian et al., 2018). When the 3D sphere size reached 300–400 μm, we initiated neural induction directly from 3D spheres while shaking by simply switching to neural induction media. To generate MCOs, we modified a recently published protocol (Figure 2A; Ormel et al., 2018) by reducing the heparin concentration to 0.1 μg/ml in the neural induction media from 1 to 10 μg/ml that is widely used for conventional CO culture (Lancaster and Knoblich, 2014; Qian et al., 2018). The dynamic morphological changes we observed during neural induction and MCO maturation (Figure 2B) follow similar patterns to that of typical CO development as widely described across various reports (Lancaster et al., 2013; Lancaster and Knoblich, 2014; Quadrato et al., 2017; Qian et al., 2018; Velasco et al., 2019). Briefly, during EB formation, MCOs begin as small, rounded spheres of generally uniform size. During neural induction, the EBs begin to form neuroepithelial tissue, characterized by a slightly translucent surface occasionally with some budding. The neuroepithelial tissue begins to elongate as radially organized structures. Embedding in Matrigel helps facilitate the growth and organization of neuroepithelial tissue as the MCOs form lobe-like structures. As MCOs continue to mature, they increase in size and maintain a spherical shape.

**Figure 3:**
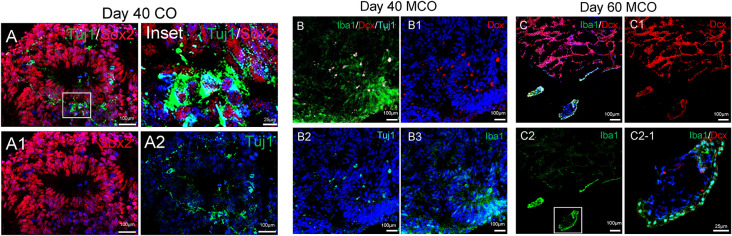
Identification of neural stem/progenitor cells (Sox2), neuroblast (Dcx), immature neurons (Tuj1), and microglia-like cells (Iba1) in developing CO and MCOs detected by multilabeled immunofluorescent confocal imaging with indicated cellular markers.** (A)** CO at day 40. **(B)** MCOs at day 40 and **(C)** at day 60. Hoechst was used as a nuclear marker. Panels **(A1–A2)**, **(B1–B3)**, and **(C1–C2)** are the split channels for **(A)**, **(B)**, and **(C)**, respectively. Panel **(C2-1)** is digital zoom-in of the inset in **(C2)**.

**Figure 4:**
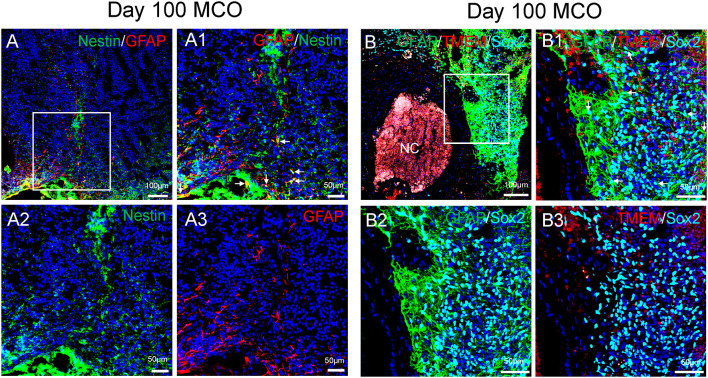
Characterization of cell types in mature MCOs. At day 100 post-EB formation, multiple cell types can be detected by multi-labeled immunofluorescent confocal image analysis with three colors **(A)** and four colors **(B)**, including NSCs (Nestin ^+^/GFAP^+^ or Sox2^+^/GFAP ^+^, arrows), NPCs (Nestin^+^ or Sox2^+^ but GFAP^−^), astrocytes (GFAP^+^), and microglia-like cells (TMEM119^+^). Hoechst was used as a nuclear marker. NC, necrotic center. Panels **(A1)** and **(B1)** are the insets from corresponding **(A)** and **(B)**. Panels **(A2/A3)** and **(B2/B3)** represent split single or double channels, respectively to **(A1)** and **(B1)**.

**Figure 5:**
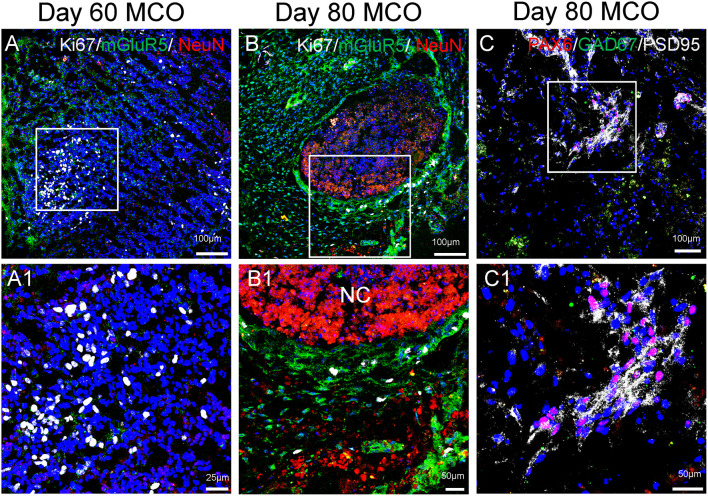
Expression of mature neurons and clusters of NPCs in MCOs. Neuronal markers are present in MCOs at days 60 and 80, as detected by multi-labeled immunofluorescent confocal analysis. **(A)** At day 60, populations of proliferating cells (Ki67^+^) and mature neurons (NeuN^+^) can be detected. Small clusters of postsynaptic excitatory receptors are detectable as well (mGluR5^+^). **(B)** At day 80, proliferating cells and mature neurons are still detectable, with mGluR5 expression appearing more pronounced and Ki67 expression appearing decreased, suggesting a trend towards neuronal differentiation vs. neural cell proliferation. **(C)** NPCs (PAX6^+^) are expressed in day 80 MCOs, as well as inhibitory GABAergic neurons (GAD67^+^) and postsynaptic proteins (PSD95^+^). Hoechst was used as a nuclear marker. NC, necrotic center. Panels **(A1)**, **(B1)**, and **(C1)** represent the digital zoom-in images from the corresponding insets.

The feature of the developing brain organoids was validated by determining the presence of neural cells within MCO using multilabeled immunostaining and confocal image analysis. Typical neuroepithelial tissues with self-organized rosette-like clusters of neural cells around ventricles were apparent in different CO/MCO regions (Figure 3). Within these clusters, populations of NSCs (co-expressing Nestin or SOX2 and GFAP) and NPCs (expressing Nestin or SOX2 but not GFAP) were evident (Figure 4). Various degrees of neural differentiation were identified by the presence of neuroblasts/immature neurons (DCX^+^ and TUJ1^+^; Figure 3), mature neuron (NeuN, Figure 5), astrocytes (GFAP, Figure 4), and others. A cluster (or aggregate) of dividing/proliferating NSCs/NPCs were identified by the presence of Ki67^+^ and PAX6^+^ immunoreactivity (Figure 5). These data suggest that our simplified protocol works well for the generation of CO or MCO with characteristic features of developing brain structures.

### Dynamic Development and Maturation of Microglia-Like Cells in MCOs

**Figure 6:**
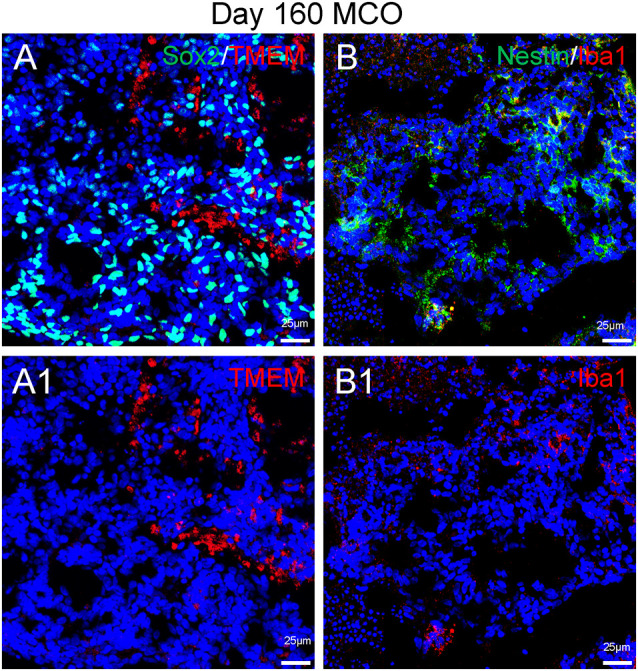
Presence of microglia in Day 160 MCOs determined by microglia-specific marker TMEM119 **(A)** and IBa1 **(B)**. Neural stem/progenitor cells were labelled with anti-Sox2 **(A)** and anti-Nestin **(B)** antibodies. Hoechst was used as a nuclear marker. NC, necrotic center. Panels **(A1)** and **(B1)** are the split channels for corresponding **(A)** and **(B)**.

**Figure 7:**
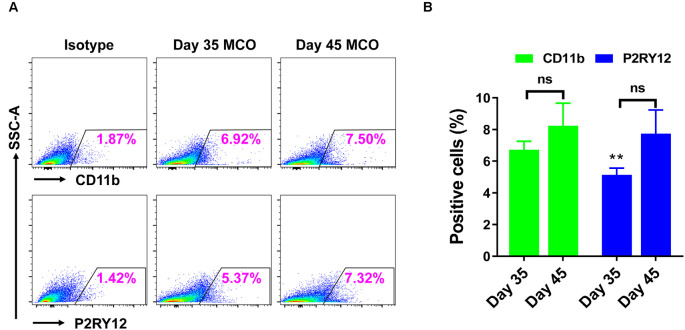
Flow cytometry analysis identified the presence of microglia in iPSC-derived MCOs. The MCOs cultured for 35 and 45 days were subjected to flow cytometry analysis with CD11b and P2RY12. Isotype controls of the corresponding antibodies were used to determine the baseline expression of microglia marker. **(A)** Representative side scatter (SSC) images of one MCO. **(B)** Diagram graph showing the average percentage of positive cells per MCO. Data represent mean ± SE of three MCOs per group. The **P < 0.01 indicates the significant difference from corresponding marker CD11b at day 35. The ns indicates no significance statistically as compared with corresponding day 35 in each marker.

Only one report demonstrated the feasibility of innately developing microglia in MCO, using a spinning bioreactor culture system (Ormel et al., 2018). To validate this report, we included part of this protocol into our simplified CO culture protocol as described above. The presence of microglia-like cells was validated by immunofluorescent staining with microglia/macrophage marker IBA1 (Figure 3) as well as microglia-specific marker TMEM119 (Figure 4). These microglia-like cells began to be detectable around day 13–16, expanded dramatically at day 30–60, and matured to the characteristic ramified morphology at day 60–180 (Figures 3, 4, 6). They scattered throughout the MCO, mainly located around epithelial layers at an early stage (Figure 3) but migrated/scattered to other regions at late stages (Figures 4B, 6). Flow cytometry analysis with CD11b and P2RY12 surface markers further confirmed the existence of microglia in average of 7 ± 2% (Figure 7), consistent with the reported number of microglia (0.5–16.6%) in the human brain, which varies with the brain region and developmental stage (Lawson et al., 1992; Nikodemova et al., 2015; Bachiller et al., 2018).

### Neuronal Maturation and Synaptic Network Formation in Mature MCOs

**Figure 8:**
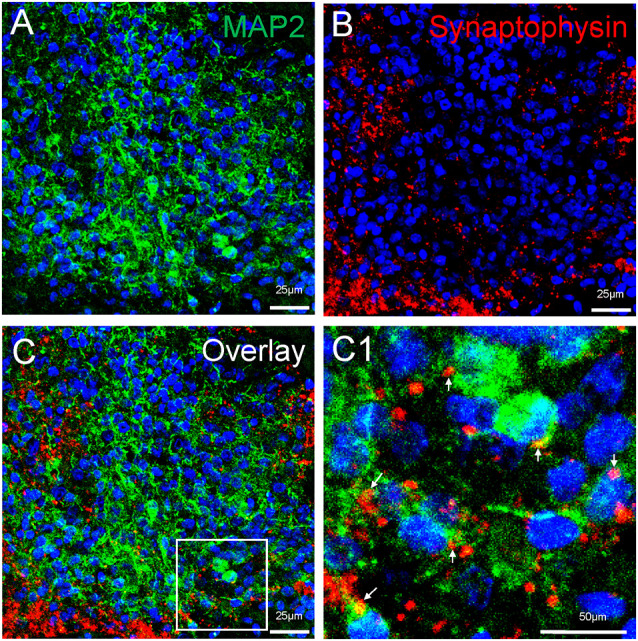
Detection of synaptogenesis in MCOs. Multi-labeled immunofluorescent and confocal analysis shows evidence of neuritogenesis/ synaptogenesis and apoptosis in MCOs. **(A,B)** Enriched dendritic arborization with long process (MAP2^+^) and presynaptic axonal terminals (Synaptophysin^+^), axon-dendritic synapse connections (arrows) were clearly visualized in all tested sections of MCOs. **(C)** represents the overlay image from **(A)** and **(B)**. Panel **(C1)** is digital zoom-in image of the inset in **(C)**.

Neuronal differentiation and maturation occur instantly during the expansion and maturation of MCOs. Mature neurons with extensive synaptic networks were detectable with neuronal markers NeuN (Figure 5) and MAP2 (Figure 8) as well as the postsynaptic protein PSD95 (Figure 5C) and presynaptic axonal marker synaptophysin (Figure 8), suggesting some regions actively undergo neuronal differentiation and functional connection. Extensive expression of the glutamatergic/excitatory neuronal marker mGluR5 was detected in most regions of MCOs at day 60–80 (Figures 5A,B), while the GABAergic/inhibitory neuronal marker GAD67 was weakly expressed at this stage (Figure 5C). More detailed analysis shows evidence of synaptogenesis/neuritogenesis in mature MCOs. Neurons display robust expression of dendritic markers (MAP2) with dendritic arborization evident in some regions (Figure 8). The presynaptic axonal terminal marker (synaptophysin) was highly expressed (Figure 8), with some visible axonal-dendritic synaptic connections (white arrows). These data suggest that mature neurons present in MCOs actively form extensive neurites, enriched dendrite arborization, and functional synaptic network.

## Discussion

Organoids have been extensively utilized to study organ developmental events and model various human diseases. However, some challenges remain, including reproducibility, predictability, scalability, cost, technical complexity, anatomical accuracy, etc. Here we design a system that simplifies the process of MCO generation from human iPSCs in an easy-to-follow and affordable protocol that can be adapted by most standard labs. Additionally, our protocol incorporates microglia to more fully recapitulate the cell types present in the brain.

There have been a great number of advances in CO technology since they were first introduced. Originally, CO generation required expensive equipment, which made it inaccessible for many labs, as well as large volumes of reagents and culture media, since spinning culture is necessary for CO to receive nutrients. Since then, protocols have simplified this process by using novel ideas to generate organoids on a smaller scale, such as the miniature spin omega bioreactor (Qian et al., 2016; Romero-Morales et al., 2019). Our protocol simply uses an orbital shaker and six-well plates in standard incubators; something that is affordable and accessible for most labs. Initial organoid protocols were also fairly complicated, involving precise technical skills such as picking iPSC colonies, generating Matrigel droplets and EB formation (Lancaster and Knoblich, 2014; Qian et al., 2016, 2018; Sloan et al., 2017; Pasca, 2018; Sutcliffe and Lancaster, 2019). Simplifying these steps may help decrease variability and increase efficiency while reducing cost and labor. Our novel protocol allows us to go directly from 2D to 3D culture, and then from 3D directly to EB culture in a six-well plate. This novel proof of concept for 3D spheres derives from previously described swirler culture for hematopoietic organoids using a bulk-cell aggregation method (Schulz et al., 2012). Our protocol also takes advantage of the newly developed mTeSR-3D suspension culture medium that allows for the expansion and scaling-up of undifferentiated human iPSCs or ESCs. The combination of these components saves time and reagents by employing a daily feeding system to replenish nutrients rather than full media changes and generates evenly distributed 3D spheres that allows direct differentiation into EBs in the same six-well plate under orbital shaking. Additionally, we adapt Qian’s method (Qian et al., 2016, 2018) of embedding multiple organoids in Matrigel in 6-well plates, which are then dissociated 4 days later for shaking culture. After this point, organoids need minimal maintenance by changing media every 3–4 days and can be continuously cultured for at least 150 days tested currently in our lab, although over 590 days for CO maintenance have been reported (Sloan et al., 2017).

Another major shortcoming of original CO modeling is the lack of diverse cell types that fully recapitulate the brain. Many studies report evidence for the presence of neurons, NSC/NPCs, and glial cells to varying degrees. However, microglial cells and endothelial cells are largely absent in original CO systems. Several protocols have been developed to co-culture iPSC-derived microglia with CO or iPSC-derived neural cells (Dos Reis et al., 2020; Fritsche et al., 2020; Hasselmann and Blurton-Jones, 2020; Tanaka and Park, 2021). While these methods are effective at generating MCOs, they require separate culturing systems with multiple additional steps as well as optimization of cell number ratio. A recent report found that simply reducing the level of heparin in the culture media during neural induction from EB could derive mesodermal microglia into the original ectodermal CO using a cost-ineffective spinning bioreactor system (Ormel et al., 2018). We combined this novel method with our direct 3D-to-EB transition technique and successfully recapitulated the generation of MCO. While microglia have traditionally been studied in the context of immune response to pathogens in the brain, they are emerging as important players in a number of neurological pathways. Importantly, microglial progenitors enter the brain very early during embryonic development and colonize among developing NSCs/NPCs (Hammond et al., 2018; Thion et al., 2018). Therefore, these microglia and neural cells differentiate and mature in close proximity to each other during development, resulting in unique cross-talk and overlapping pathways. For example, microglia have been shown to regulate the number of NSCs/NPCs as well as play roles in postnatal presynaptic pruning and synaptogenesis (Cunningham et al., 2013; Miyamoto et al., 2016; Weinhard et al., 2018). However, some of these mechanisms have not fully been explored during early neurodevelopment, but MCOs may present a unique model to study these critical interactions, particularly in the context of elucidating mechanisms underlying neurodevelopmental disorders and neurodegenerative diseases. Furthermore, as microglia are known to play critical roles in inflammation and infection, MCOs provide an excellent model for neurotropic viral infections such as HIV and ZIKV (Muffat et al., 2018; Dos Reis et al., 2020; Qiao et al., 2020; Bodnar et al., 2021). While organoids have been used to study viral infections before (Cugola et al., 2016; Dang et al., 2016; Qian et al., 2016; Gabriel et al., 2017; Li et al., 2017b; Watanabe et al., 2017), these models did not include microglia, which are critical to the immune response.

One limit of this study is the small number of microglia cells in MCO (around 7%), although it may represent the physiological development process (Lawson et al., 1992; Nikodemova et al., 2015; Bachiller et al., 2018). Optimization of the heparin concentration or other regulators may improve the generation of microglia in MCO. Another strategy might be the co-culture of iPSC-derived microglia with the CO (Dos Reis et al., 2020; Rai et al., 2020), which may increase the number of microglia cells in CO, although the process does not mimic normal brain development. Another limit of our MCO model is that there are still several cell types missing, including endothelial cells/vasculature. While vasculature is an integral part of the brain, it was initially difficult to incorporate into the organoid culture, with earlier protocols requiring the use of compartmentalized microfluidic chips for the introduction of endothelial cell co-culture (Nashimoto et al., 2017; Grebenyuk and Ranga, 2019) or even grafting cerebral organoids *in vivo* in mouse brains, which vascularize over time and form functional circuits (Daviaud et al., 2018; Mansour et al., 2018). However recent publications have used novel ways to incorporate endothelial cells by co-culture with mesodermal cells (Wörsdörfer et al., 2019, 2020; Shi et al., 2020) or innate induction *via* inducible expression of ETV2 (Cakir et al., 2019). We anticipate that some of these methods can be incorporated into our MCO protocol for future experiments and the development of a more complete model of neurodevelopment. However, the vasculature in these organoid models is not fully mature as there is no blood flow, so doing so would require either *in vivo* grafting into a host (Mansour et al., 2018) or incorporation of a microfluidic system (Wörsdörfer et al., 2020). Finally, it is important to note that the MCOs produced are not region-specific and therefore representative of general cerebral cortex tissue, with morphology similar to that of traditional organoid protocols (Lancaster and Knoblich, 2014). It may be possible to incorporate the microglia-promoting components of our protocol with that of region-specific protocols for more advanced organoid models/brain assembloids (Qian et al., 2018; Sloan et al., 2018; Xiang et al., 2020), but would require further optimization due to differences in neural induction and patterning techniques.

In summary, we present a novel protocol that allows for the simplified generation of cerebral organoids that contain microglia (MCO). This protocol can be readily adapted by most standard labs and scaled as needed. Additionally, immunohistochemistry and confocal image analysis shows the expression of NSCs/NPCs/neurons as well as astrocytes and microglia at various time points, showing expected characteristics of organoids/developing brain making this a viable model to use for the investigation of human brain development and pathogenesis in various diseases. By simplifying the process of generating organoids, it makes this novel and exciting tool more accessible for labs, which will ultimately help further innovations in this field in a collaborative way to generate reproducible organoids.

## Data Availability Statement

The raw data supporting the conclusions of this article will be made available by the authors, without undue reservation.

## Author Contributions

WH, YZha, QL, and W-ZH contributed to the conception and design of the study. BB, YZha, JL, YL, PW, ZW, SS, YZhu, FL, and XW conducted the experiments and acquired data. BB and WH analyzed the data and wrote the manuscript. WY and YZhu generated iPSC. All authors contributed to the article and approved the submitted version.

## Conflict of Interest

A U.S provisional patent application related to this work was filed with WH as the inventor. The remaining authors declare that the research was conducted in the absence of any commercial or financial relationships that could be construed as a potential conflict of interest.

## Abbreviations

3D: three-dimensional
CO: cerebral organoid
EB: embryoid body
hESC: human embryonic stem cell
iPSC: induced pluripotent stem cell
MCO: microglia-containing cerebral organoid
MEF: mouse embryonic fibroblast
NPC: neural progenitor cell
NSC: neural stem cell.
